# Financial Factors and Psychological Distress during the COVID-19 Pandemic in Poland

**DOI:** 10.3390/ijerph19031798

**Published:** 2022-02-05

**Authors:** Katarzyna Sekścińska, Agata Trzcińska, Daniel Pankowski, Ewa Pisula, Kinga Wytrychiewicz-Pankowska

**Affiliations:** 1 Faculty of Psychology, University of Warsaw, 00-183 Warsaw, Poland; atrzcinska@psych.uw.edu.pl (A.T.); daniel.pankowski@psych.uw.edu.pl (D.P.); ewa.pisula@psych.uw.edu.pl (E.P.); kwytrychiewicz@gmail.com (K.W.-P.); 2 Institute of Psychology, University of Economics and Human Sciences in Warsaw, 01-043 Warsaw, Poland

**Keywords:** psychological distress, depression symptoms, anxiety symptoms, financial variables, COVID-19 pandemic

## Abstract

During the COVID-19 pandemic, many factors have simultaneously affected people’s psychological distress (PD). The most commonly studied types of factors have been those relating to health risks involving SARS-CoV-2 infection and sociodemographic factors. However, financial changes at both the national and global levels and these changes’ influences on people’s personal finances constitute another group of factors with the potential to cause symptoms of anxiety and depression. A correlation study of 1135 working adults in Poland was conducted to analyze the roles of a wide range of financial variables in explaining the extent of people’s PD during the pandemic. Three groups of financial factors predicted PD over and above sociodemographic variables and COVID-19 health-related factors: a person’s objective financial situation, their subjective financial situation, and their individual financial disposition, the last of these being the most important. The present study adds to the current state of knowledge by showing that financial variables explain a significant portion of variance in PD over and above sociodemographic and COVID-19 health-related factors. Moreover, the study also identified individual financial variables that were capable of predicting people’s psychological distress during the pandemic.

## 1. Introduction

The COVID-19 pandemic has had a significant impact on almost all areas of people’s lives. In addition to the threat it has posed to people’s physical health and lives, it has been associated with a number of other burdens. In response to the developing health threat, governments have introduced numerous measures, such as social distancing, closing borders, banning gatherings, closing selected industries, and mandating remote learning and working. Moreover, the circumstances surrounding the pandemic have directly and indirectly affected people’s mental health. It has already been shown that the pandemic has increased psychological distress (PD) [1,2], which is commonly defined as a state of emotional suffering characterized by symptoms of depression and anxiety [3]. The stress–distress model postulates that PD occurs when a stressful event threatens a person’s physical or mental health and they are unable to cope with the stressor effectively, emotional discomfort being the result of this ineffective coping [4]. The COVID-19 pandemic has been one such stressful event. A meta-analysis conducted by Cénat et al. [1] showed depression to be over three times more prevalent during the pandemic (occurring in 15.97% of people) than it was prior to the pandemic (4.4% of people) and the prevalence of anxiety to be four times higher (15.15% compared to 3.6%). Given these observations, to understand the pandemic’s effects on PD, it is important to determine the factors that may provide protection or constitute a threat with respect to people’s susceptibility to experiencing symptoms of depression and anxiety.

During the pandemic, many different factors may have had a simultaneous influence on psychological distress. Previous research has shown that a higher infection risk (e.g., the number of suspected/confirmed COVID-19 cases in a geographical location) and increased risk of developing a severe infection (having a pre-existing physical condition or worse general health) are predictive of a greater severity of PD [5]. Experience with COVID-19 is also an important risk factor for PD, and a study of Cai [6] showed that the severity of PD is particularly high in people who have developed COVID-19. 

Moreover, other research has identified the important roles of sociodemographic variables such as gender, age, place of residence, and educational level in explaining people’s tendency to experience symptoms of depression and anxiety [5,7,8,9]. Studies have indicated that women have been at increased risk of developing psychopathological symptoms during the pandemic [7,10] and that this may be related to the fact that they are more likely than men to work in positions with a high risk of SARS-CoV-2 infection (the virus that causes COVID-19), e.g., nurses, shop assistants, kindergarten teachers, beauticians, and hairdressers. Previous work also showed that being of an older age was protective against psychological distress during the pandemic [11], and it is likely that losing a job and/or uncertainty resulting from the restrictions introduced may have been particularly stressful for people under 40 years of age due to the increased likelihood of them being caretakers of children or older family members. Moreover, emerging adults (18–29 year olds) may have been particularly at risk of PD, because mandatory social distancing, isolation from people outside their household, and restrictions on movement and travel are likely to have interfered with their need to build their autonomy and maintain friendships and romantic relationships [8]. Place of residence is a further sociodemographic factor that has been connected with PD during the pandemic, the research indicating that people living in rural areas have been at greater risk of PD compared to those living in urban areas [5]; this may be because of inferior medical, economic, and educational infrastructure in rural areas [12]. Another factor that may be indirectly related to the greater incidence of PD in people living in rural areas is socioeconomic status, including lower levels of education; the meta-analysis of Wang et al. [5] showed that less well-educated people are likely to have been at risk of developing symptoms of depression and anxiety during the pandemic.

As shown above, both COVID-19 health-related variables (number of suspected/confirmed COVID-19 cases and increased risk of developing severe COVID-19) and sociodemographic variables have been extensively investigated as predictors of psychological distress during the pandemic. However, despite the fact that the restrictions introduced by governments during the pandemic have had significant negative financial consequences for many people, and the likelihood that this will have been the cause of increased psychological distress in such people, there has been a lack of research identifying the extent to which various financial variables can augment sociodemographic and health threat-related variables in explaining the psychological distress that has occurred during the pandemic. The present study aimed to fill this gap. As in other countries, high levels of anxiety and depression symptoms were observed in Poland (where our study was conducted) during the pandemic [8]. Given the potential long-term consequences of temporarily elevated psychological distress, it is important to determine factors other than the commonly studied COVID-19 health-related variables and sociodemographic variables that may have contributed to or been protective against anxiety and depression.

### 1.1. Financial Variables and Psychological Distress

As mentioned above, the coronavirus pandemic has increased anxiety and depression symptoms among people across the globe. This may be partly due to the effects that government-imposed restrictions have had on countries’ economies and workforces (for example, people’s income and job security—see, e.g., Reference [13]). A number of studies have shown that various financial factors can be associated with psychological distress [14,15,16] and that people’s mental health may be associated both with their objective financial situations (income, level of savings, and level of liabilities including debt—e.g., References [17,18,19] and their subjective perceptions of their financial situations (perceived income, sense of financial security, and job security—e.g., References [13,14,20]. Additionally, studies have shown that psychological distress may be associated with individual financial dispositions such as materialism, economic optimism, and the propensity to take financial risks [21,22,23].

#### 1.1.1. Objective Financial Situations and Psychological Distress

The effect that a household’s objective financial situation can have on the risk that its members will experience anxiety and depression is well-documented [15,24,25]. For example, Orpana et al. [16] demonstrated that a lower income is associated with a higher risk of becoming psychologically distressed. Additionally, using British Household Panel Survey data, Wildman [19] found that a person’s financial situation and changes in their situation to be associated with depression, and conversely, research conducted in Sweden has shown that a high household income may be protective against symptoms of depression [26]. Moreover, Gambin et al. [8] showed that, during the COVID-19 pandemic, continuity in Polish people’s incomes and their financial situations has had a significant effect on experiencing symptoms of anxiety and depression.

In addition to household income, the objective level of a person’s debt has also been shown to be related to their mental health. Brown, Taylor and Wheatley Price [27] observed that debt is associated with increased levels of psychological distress and that a household’s psychological well-being is adversely affected by large amounts of unsecured debt (but not secured debt, such as a mortgage on a house). Additionally, there is a positive relationship between the amount of debt and the experiencing of mental health problems [18], and both Skapinakis et al. [28] and Jenkins et al. [29] showed that excessive debt and problems in paying it off make it more likely that a person will become depressed. Finally, Drentea [30] showed that anxiety increases as the ratio of credit card debt to income increases. 

At the same time, researchers have emphasized that savings can act as a type of buffer against psychological distress. For example, Brown, Taylor and Wheatley Price [27] showed that people who save (or whose households save) on a regular basis are more likely to report complete psychological well-being than non-savers. Additionally, Bridges and Disney [17] noted that the number of savings accounts that a person had (which was associated with having greater savings) was indirectly associated with a lowered incidence of self-reported symptoms of depression. Moreover, Gardner and Oswald [31] found that positive ‘shocks’ (medium-sized lottery wins) to the financial well-being of a household tended to be associated with improvements in psychological well-being.

#### 1.1.2. Subjective Financial Situations and Psychological Distress

Bridges and Disney [17] noted that objective indicators of a household’s financial situation are not sufficient in understanding the impact of economic factors on psychological distress. These authors found that positive relationships between depression symptoms and self-reported problems of indebtedness and financial stress arise irrespective of objective indicators of a household’s financial situation. Thus, when investigating economic sources of psychological distress, one should pay attention not only to the objective financial factors but also to how individuals perceive their financial situation. In fact, research conducted as long as forty years ago showed that perceptions of economic hardship in acquiring the necessities of life dispose people toward depression [32], and more recent research by Dijkstra-Kersten et al. [14] showed financial strain to be associated with having a depressive and/or anxiety disorder, over and above the effects of income.

O’Neill et al. [33] noted that people are happier when they are financially secure, and Ferrie et al. [20] showed that differences in self-reported financial insecurity are important determinants of differences in the incidence of depression. The COVID-19 pandemic has caused many people to worry more about their financial security and experience greater financial concerns, and the research by Wilson et al. [13] showed that, among people saying that the pandemic is posing a significant threat to their personal finances (e.g., those expecting their financial situation to worsen over the next 12 months), 57% of people reported experiencing moderate and high levels of psychological distress, including symptoms of depression and anxiety. Furthermore, it should be noted that, while a sense of financial security and financial concerns are largely related to job security, perceived job insecurity is a stressful experience in itself and is related to greater symptoms of depression and anxiety, as shown by Wilson et al. [13].

#### 1.1.3. Individual Financial Dispositions and Psychological Distress

The studies described above show that psychological distress may depend on both a person’s objective financial situation and how they perceive their financial situation. However, many previous studies have shown that an individual’s mental health is related not only to their financial situation (whether objective or subjective) but also to their individual financial dispositions: their psychological characteristics with respect to financial issues [34]. Examples of these include materialism, economic optimism, and the tendency to take financial risks. Probably, the most frequently analyzed financial disposition in the context of well-being and psychological distress is materialism, this being defined as “the importance a person places on possessions and their acquisition as a necessary or desirable form of conduct to reach desired end states, including happiness” [35] (p. 307). Materialism has been linked to mental health problems, particularly depression and anxiety [23,36,37,38], and a meta-analysis of several hundred studies by Dittmar et al. [39] showed clear negative relationships between a broad array of types of personal well-being (including depression and anxiety) and people’s materialistic values. 

In addition to materialism, a second financial disposition that is associated with PD is economic optimism. Optimists see positive aspects of current situations and events and believe that things will turn out to be positive in the future as well. One recent study of people’s economic optimism (often referred to as consumer confidence or consumer sentiment) by van Giesen and Pieters [40] recently showed that the more optimistic people are about economic issues, the less personal stress they experience, this applying irrespective of whether the economic issues involved are on a global, national, or personal level. Additionally, research by Kahle et al. [21] showed that economic optimism is negatively associated with depression.

Another financial disposition that may be related to psychological distress is a propensity for financial risk-taking. Here, studies have shown that risk avoidance is associated with high anxiety [22,41]. In these studies, and in other studies on emotions and risk-taking [42,43], researchers have assumed that emotions such as anxiety trigger risk-averse behaviors. However, given that a propensity for risk-taking is strongly rooted in personality [44] and is a relatively constant individual trait, the relationship between a propensity for financial risk-taking and emotional variables is probably bidirectional. Such an assumption is consistent with the results of Gutter and Copur [34], who showed that an unwillingness to take any financial risks at all is negatively related to financial well-being (which is likely to be related to general well-being). Moreover, in this study, people characterized by a greater than average willingness to take financial risks reported higher levels of financial well-being. This suggests that a higher propensity to take financial risks may translate into lower levels of psychological distress.

In summary, all the above studies of the role of economic variables in generating psychological distress provide evidence that financial factors may be important in explaining PD during the COVID-19 pandemic and that such factors are worth considering in the search for factors that may have a protective function or present a threat with respect to people’s susceptibility to experiencing symptoms of depression and anxiety. Due to the deteriorating economic situation, it can be assumed that the financial effects of the pandemic will affect more and more people over time and be one of the most important sources of PD in the short- to medium-term future.

### 1.2. The Current Study 

During the COVID-19 pandemic, financial changes on both the global and national scale have affected many people’s personal finances, and this is likely to have caused people to experience symptoms of anxiety and depression. Therefore, one of the present study’s main goals was to explore whether financial variables could explain the variance in people’s psychological distress (PD) over and above the sociodemographic and COVID-19 health-related variables.

The study’s second aim was to ascertain which financial factors are the most important predictors of PD. While previous studies have established links between various financial factors and PD, these studies have tended to analyze each financial variable in isolation without simultaneously analyzing the financial status (both objective and subjective) and financial disposition variables’ relationships with symptoms of anxiety and depression. Therefore, to date, studies have been unable to determine the relative importance of various financial factors in predicting PD, and this is particularly important in the context of the COVID-19 pandemic. Thus, our research considered a wide spectrum of financial factors as predictors of PD during the pandemic. We analyzed the role of financial factors on three levels, which allowed us to answer three research questions: (1) Taken together, can financial variables explain the variance in the levels of people’s depression and anxiety symptoms over and above the variance explained by sociodemographic and COVID-19 health-related factors? (2) Which category of financial factors (people’s objective financial situation, their subjective financial situation, or their individual financial dispositions) explains the largest proportion of variance in PD symptoms? (3) Which specific financial variables in the aforementioned three categories are the strongest predictors of variance in PD symptoms?

## 2. Materials and Methods

### 2.1. Participants

A nationwide sample of working age (18+ year olds) Polish adults, diversified in terms of age, sex, place of residence, and educational level, were recruited online from the ARIADNA research panel. ARIADNA is a Polish online research panel with over 150,000 registered Polish users. For any particular study, ARIADNA randomly recruits voluntary participants from its panel (registered users), collects data, and then provides the anonymized data to the party conducting the research. Respondents were awarded points for their participation, which they could later exchange for rewards from a pool of several hundred products offered by ARIADNA. Panel studies may have some limitations, for example: the incentive structure makes web panels more attractive to low-income respondents, research is limited to internet users, and data integrity concerns may arise. Nevertheless, the sociodemographic profiles of people registered in the ARIADNA panel corresponds with the profiles of Polish Internet users, and recruitment to the panel is carried out continuously. The panel has a current and valid Interviewer Quality Control Programme (PKJPA) certificate confirming the high quality of the research services provided, issued on the basis of an independent audit carried out annually by the Polish Association of Public Opinion and Marketing Research Firms. Only genuine persons of a verified identities participate in research on the ARIADNA panel. 

A total of 1135 working people (732 women and 403 men, aged between 18 and 87 years-old; M = 38.68 years, SD = 11.59 years) participated in the study. More detailed socioeconomic data for the participants are presented in Table 1. A sensitivity analysis using G*Power [45] revealed that the sample provided 80% power for detecting effect sizes of *f*^2^ = 0.01 to *f*^2^ = 0.02 (depending on the model tested).

### 2.2. Materials

#### 2.2.1. Psychological Distress

*Anxiety symptoms*. Participants’ current levels of anxiety symptoms were measured using a Polish version (MAPI Institute; www.phqscreeners.com) of the General Anxiety Disorder—7 scale (GAD-7) [47]. This self-rating questionnaire consists of seven items that have response options ranging from “not at all” to “nearly every day”. The total scores range from 0 to 21, with higher scores reflecting greater anxiety. The scale was shown to exhibit high reliability in the present study, the Cronbach’s α being 0.95.

*Depression symptoms*. Symptoms of depression were measured by a Polish version (MAPI Institute; www.phqscreeners.com) of the Patient Health Questionnaire—9 (PHQ-9) [48]. This self-rating questionnaire contains nine items measuring the severity of symptoms of depression. Each item is scored on a four-point Likert scale ranging from 0 (not at all) to 3 (nearly every day). A total score is computed by summing the scores for all the items, with higher scores reflecting a greater severity of depression. A Cronbach’s α of 0.93 revealed a high reliability for the present study.

#### 2.2.2. Financial Variables

Sixteen financial variables were included in the study. These variables fell into three categories: objective financial situation, subjective financial situation, and individual financial dispositions. 

Objective financial situation variables:

*Monthly net income per capita for household*—participants indicated their net household income on the following scale: 1—lower than PLN 1000~USD 264; 2—PLN 1001 to 2000~USD 265 to 528; 3—PLN 2001 to 3000~USD 529 to 792; 4—PLN 3001 to 4000~USD 793 to 1056; 5—PLN 4001 to 5000~USD 1057 to 1320; 6—>PLN 5000~>USD 1320.

*Possession of savings* was measured using one question where participants indicated whether they currently had (answer “yes”) or did not have (answer “no”) savings.

*Saving money before the COVID-19 pandemic* was measured using one question where participants indicated whether they had saved money during the last six months before the COVID-19 pandemic (answer “yes”) or not (answer “no”).

*Financial liabilities* were measured using two questions: one relating to the holding of loans/credit (scaled yes/no) and one concerning the size of monthly commitments relating to any such loans/credits (measured as the percentage of monthly household income allocated to debt repayments and scaled as follows: 1–15%, 16–30%, 46–65%, 66–80%, 81–95%, and more than 95%).

Subjective financial situation variables:

*Subjective assessment of the current household financial situation*—participants answered the following question: How do you rate the current financial situation of your household? To answer, they provided a rating on a scale from 1 (very bad) to 7 (very good).

*Subjective change in household financial situation*—measured by asking participants to give a subjective assessment of the financial change in their household’s situation during the COVID-19 pandemic on a scale from 1 (significantly deteriorated) to 7 (significantly improved). 

*Financial security*—participants answered the following question: How financially secure do you feel today? They responded on a scale from 1 (definitely insecure) to 7 (definitely secure).

*Perceived job security*—participants answered the following question: In the current pandemic situation, can you be sure of keeping your job? Possible responses were “yes” and “no”.

Individual financial dispositions: 

*Materialism*: general materialism was measured using the short version of the Material Values Scale (MVS) developed by Richins and Dawson [35]. The short version of the MVS is a three-dimensional self-reporting measure consisting of nine statements to which answers are given on a scale from 1 (definitely no) to 5 (definitely yes). The instrument provides a total score (general materialism) and subscale scores: centrality (e.g., I like a lot of luxury in my life), happiness (e.g., I’d be happier if I could afford to buy more things), and success (e.g., The things I own say a lot about how well I’m doing in life). Presently, only a total general materialism score was computed by summing participants’ responses to all nine items. Richins [49] conducted a meta-analysis of 15 studies, thus confirming the reliability and empirical utility of the MVS. The tool’s reliability was acceptable in the present study (Cronbach’s α = 0.83).

*A general propensity to take financial risks* was measured using two subscales of the DOSPERT scale [50]. This scale consists of 30 statements relating to four different risk domains: ethical, financial (divided into gambling and investing subdomains), health/safety, and social. In the present study, only the two financial subscales were used. Each of these subscales is comprised of three items (e.g., Betting a day’s income at the horse races, and Investing 5% of your annual income in a very speculative stock). Participants were asked to indicate the likelihood that they would engage in the described activity or behavior on a scale from 1 (very unlikely) to 7 (very likely). An indicator of a general propensity to take financial risks was calculated as the sum of the answers given for all six DOSPERT scale items used. For the present data, the measuring was found to exhibit a high degree of reliability (Cronbach’s α = 0.91).

*Economic optimism* was measured on both the national and household levels, and with respect to both short (6 months) and long (18 months) periods, using four questions: Questions 1 and 2 asked participants—How do you think the situation brought about by the pandemic will affect the economic situation in the country at the end of this year (December 2020)/at the end of next year (December 2021) in relation to the economic situation before the outbreak of the pandemic? Questions 3 and 4 asked participants—How do you think the situation brought about by the pandemic will affect your household financial situation at the end of this year (December 2020)/at the end of next year (December 2021) relative to your financial situation before the outbreak of the pandemic? Participants were asked to indicate their answers on a scale from 1 (it will definitely deteriorate) through 4 (it will remain unchanged) to 7 (it will definitely improve).

#### 2.2.3. COVID-19 Health-Related Variables

*COVID-19 experience*—this variable was coded 1 for participants with experience of COVID-19 and 0 for participants with no experience of COVID-19. Having experience of the disease was measured using four questions: Are you/have you been infected with coronavirus? Has anyone close to you been diagnosed with the coronavirus infection? Does anyone close to you have, or have they had, symptoms of coronavirus infection? Are you, or have you been, subject to home quarantine for suspected coronavirus infection? Participants were considered to have experience of COVID-19 if they answered “yes” to at least one of these questions. 

*Risk grouping for severe COVID-19*—participants were placed into two groups depending on whether they had a medical condition that would be likely to place them at risk of developing severe COVID-19 symptoms if they were to become infected with SARS-CoV-2. Participants giving a positive answer to at least one of the following questions were coded as being at severe risk: Do you suffer from a chronic disease? Are you on immunosuppressive therapy or chemotherapy? Do you need ongoing medical care (e.g., for pregnancy, chemotherapy, dialysis, etc.)? Participants not answering positively to any of these questions were coded as not being at severe risk.

#### 2.2.4. Sociodemographic Data 

The sociodemographic questionnaire collected data concerning participants’ gender (male/female); age (in years); place of residence (countryside, a small town of less than 20,000 inhabitants, a medium town of 20,000–99,000 inhabitants, a large city of 100,000–500,000 inhabitants, or a very large city of more than 500,000 inhabitants); and the highest level of educational attainment (primary education, secondary education, post-secondary education, bachelor/engineer degree, or Master’s degree).

### 2.3. Procedure

The study was conducted using CAWI methodology to collect data from members of an online panel. Participants completed each of the abovementioned research tools in a rotated order. Data were collected during the first wave of the COVID-19 pandemic in Poland over the period from 5 to 12 May 2020. Previous studies conducted both during the COVID-19 pandemic [51] and during earlier outbreaks of high-risk infectious diseases such as SARS (as caused by SARS-CoV-1), Ebola, and H1N1 influenza [52] showed that psychological distress is at its greatest at the very beginning of a pandemic. Over time, and despite increasing numbers of COVID-19 cases, the degree to which people have exhibited daily preoccupation with topics involving the disease and the pandemic has been shown to have decreased, resulting in decreased subjective perceptions of risk [51]. Thus, the first wave of the pandemic seems to be an appropriate point at which to analyze the pandemic’s short-term consequences for people’s mental health and to investigate factors that may have a protective function or present a threat with respect to people’s susceptibility toward experiencing symptoms of depression and anxiety. Up to the start of the study (May 5, 2020), a total of 14,006 SARS-CoV-2 infections and 698 COVID-19 deaths had been confirmed in Poland. At the time the data were collected, the daily number of new Polish cases ranged from 285 to 425 [53], and a study of psychological distress conducted during this time showed that such distress was at a high level among Poles [5]. 

## 3. Results

Since they refused to answer questions measuring the monthly net per capita income for their household or the size of their monthly commitments relating to the repayment of any loans/credits, 138 participants were excluded from the analyses. As a result, data for 977 participants were analyzed. Descriptive statistics (means and standard deviations) and zero-order correlations for the analyzed variables are presented in Appendix A (Table A1 and Table A2).

### 3.1. The Specific Role of Financial Variables (in General and When Divided into Three Categories) in Explaining Psychological Distress

Initial hierarchical regression analyses were performed to determine the specific contributions of financial variables in explaining variance in psychological distress (PD) over and above sociodemographic and COVID-19 health-related variables. Further hierarchical regression analyses evaluated the specific contributions of each category of variables in turn (sociodemographic and COVID-19 health-related variables, objective financial variables, subjective financial variables, and individual financial dispositions) in explaining the variance in PD while all the other categories of variables were controlled. Analyses were conducted for depression symptoms and anxiety symptoms as two separate dependent variables (DVs).

First, two hierarchical regression analyses were conducted, one each for anxiety and depression symptoms as DVs. In both analyses, independent variables (IVs) were introduced in the following blocks: Block 1—sociodemographic variables (gender (with females coded as 1), age, place of residence, and educational level); Block 2—COVID-19 health-related variables (COVID-19 risk grouping (with high risk coded as 1) and COVID-19 experience (with experience coded as 1)); Block 3—objective financial situation: monthly net per capita income for household, possession of savings (with possession of savings coded as 1), saving money before the COVID-19 pandemic (with having saved money coded as 1), loan/credit holding (with holding of debt coded as 1), and amount of monthly commitments related to loans/credits; Block 4—subjective financial situation: subjective assessment of current household financial situation, subjective change in financial situation, financial security, and perceived job security; Block 5—individual financial dispositions: materialism, general propensity to take financial risks, economic optimism at a household level (two variables: short-term and long-term optimism) and economic optimism at a national level (two variables: short-term and long-term optimism).

Table 2 presents the results of the hierarchical regression analyses for the two psychological distress DVs and shows cumulative statistics for all the IVs included in the models at a given step. These results indicated that financial variables made statistically significant contributions to explaining the variance in each PD measure. With sociodemographic variables and COVID-19 health-related variables already included in the models, financial variables accounted for around 15 percentage points of the additional variance in both depression and anxiety symptom scores. Moreover, the results showed that individual financial dispositions accounted for around 10 percentage points of additional variance in the two DVs over and above the sociodemographic variables, COVID-19 health-related variables, and participants’ objective and subjective financial situations.

To obtain a wider picture of the relationships between the variables analyzed, eight additional hierarchical regression analyzes were performed in which, in turn, each variable category not entered last in the first two analyses was entered last to determine the specific contributions that each variable category made to explaining variance in the two PD DVs over and above the other categories. The specific contributions made by the different categories of variables were as follows:

- sociodemographic variables: anxiety symptoms (*R*^2^ change = 0.039, *p* < 0.001); depression symptoms (*R*^2^ change = 0.045, *p* < 0.001).
- COVID-19 health-related variables: anxiety symptoms (*R*^2^ change = 0.009, *p* = 0.003); depression symptoms (*R*^2^ change = 0.009, *p* = 0.004).
- objective financial situation variables: anxiety symptoms (*R*^2^ change = 0.006, *p* = 0.235); depression symptoms (*R*^2^ change = 0.005, *p* = 0.308).
- subjective financial situation variables: anxiety symptoms (*R*^2^ change = 0.029, *p* < 0.001); depression symptoms (*R*^2^ change = 0.027, *p* < 0.001).

From the results of these analyses, it can be concluded that, relative to the roles played by the other two groups of financial variables, individual financial dispositions played the most important role in explaining the extent of people’s depression and anxiety symptoms (when sociodemographic and COVID-19 health-related variables were controlled).

### 3.2. The Specific Role of Each Financial Variable in Explaining Psychological Distress

Next, two stepwise regression analyses were conducted to identify significant individual predictors of the two psychological distress DVs. Here, all the IVs entered in the previous analyses were entered in one block. Table 3 presents the final models for each of the DVs (only significantly predictive IVs are included in the table; full descriptions of the stepwise regression analyses are presented in Appendix A: Table A3 and Table A4). 

Eight financial variables were identified as significant predictors of anxiety symptom severity: materialism, financial security, general propensity to take financial risks, short-term economic optimism at a national level, long-term economic optimism at a household level, perceived job security, and amount of monthly commitments related to the loans/credits. Financial security, perceived job security, and long-term economic optimism at the household level were negative predictors, while the other variables were positive predictors. Moreover, nonfinancial variables such as age, gender, and belonging to the group of people at severe risk from COVID-19 were also significant predictors of anxiety symptoms, age being the only negative predictor. 

Six economic variables were also identified as significant predictors of severity of depression symptoms. Materialism, general propensity to take financial risks, and short-term economic optimism at a national level were positively predictive, while financial security and long-term economic optimism at a household level were negatively predictive. Two sociodemographic variables (gender and age) and one COVID-19-related variable (belonging to the group of people at severe risk from COVID-19) were also significant predictors of depression symptoms. Among these, only age was negatively predictive.

## 4. Discussion

The study analyzed the role of financial factors in explaining psychological distress during the COVID-19 pandemic. To obtain a detailed picture, we considered two outcome variables: levels of depression and anxiety symptoms. With respect to financial factors, we took into account a wide range of variables in three categories: people’s objective financial situations, their subjective financial situations, and their individual financial dispositions. This approach to financial factors allowed us to answer three questions concerning relationships between economic variables and psychological distress: (1) Taken together, can financial variables explain variance in the levels of people’s depression and anxiety symptoms over and above the variance explained by sociodemographic and COVID-19 health-related factors? (2) Which category of financial factors (people’s objective financial situation, their subjective financial situation, or their individual financial dispositions) explains the largest proportion of variance in PD symptoms?; (3) Which specific financial variables are the strongest predictors of the variance in PD symptoms?

The results obtained showed that financial variables made a statistically significant contribution to explaining the variance in both PD variables over and above sociodemographic and COVID-19 health-related variables. While all three financial variable categories (objective financial situation, subjective financial situation, and individual financial dispositions) played an important role in explaining people’s depression and anxiety symptoms, the role of individual financial dispositions was identified as the most crucial. Individual financial dispositions accounted for around 10% of the variability in depression and anxiety symptoms over and above the variance accounted for by the sociodemographic and COVID-19 health-related variables and people’s objective and subjective financial situations, while (when they were placed in the last block in hierarchical regression analyses) the specific contributions to explaining variance in both PD indicators made by the other financial variable categories were nonsignificant in the case of an objective financial situation and relatively small in the case of a subjective financial situation (2.9% for anxiety symptoms and 2.7% for depression symptoms).

It is worth underlining that the lists of significant predictors of depression and anxiety symptoms are almost the same. While the present methodological design does not permit causal inferences to be made, higher levels of materialism, a general propensity to take financial risks, and short-term economic optimism at a national level may promote depression and anxiety symptoms, while high levels of financial security and long-term economic optimism at a household level may decrease depression and anxiety symptoms and, thus, may be protective factors. Perceived job security (being sure of keeping one’s job) and monthly loan/credit repayment amount were the only two predictors that were significantly predictive for anxiety but not for depression, with perceived job security being a potential protective factor and monthly repayment commitments being a factor that may promote anxiety. Additionally, the roles of sociodemographic and COVID-19 health-related variables in explaining the extent of people’s depression and anxiety symptoms were similar, with age being a negative predictor (and a possible protective factor) and female gender and belonging to the group of people being at severe risk from COVID-19 being positive predictors. 

Most of the preset results for particular financial variables as predictors of PD are in line with those of previous studies [5,13,17,18,23]. However, two results contradict previous studies. The first is related to a propensity for financial risk-taking. While previous studies have shown a negative relationship between financial risk-taking and anxiety symptoms [22,41], we found a financial risk-taking propensity to be a positive predictor of PD during the pandemic. This inconsistency may result from certain mediating factors arising during the pandemic. For example, people prone to taking financial risks might have made risky financial decisions before the pandemic (e.g., they might have made investments), which, because of the pandemic, turned out to be unprofitable or even financially damaging. Any such deteriorations in people’s financial situations would be likely to be positively corelated with intensity of depression and anxiety symptoms. Of course, this explanation is speculative, and further research taking into account people’s wider financial circumstances is required to obtain a better understanding of the present result.

The second result that is inconsistent with previous research concerns economic optimism. Previous studies have shown that more economically optimistic people experience less stress and exhibit lower levels of depression symptoms [21,40]. Our study showed that, although long-term economic optimism at a household level was negatively predictive of PD, short-term economic optimism at the national level was positively predictive of PD. The results of research by Xie et al. [54] on general optimism and PD during the SARS-CoV-1 pandemic may explain this pattern of results. This research showed that, while optimism can be associated with lower PD, it is also associated with greater pandemic-related vigilance, which, in turn, translates into higher levels of anxiety. Therefore, optimistic people may focus more on a pandemic and, thus, experience greater psychological distress. However, further research is required to obtain a better understanding of why economic optimism at household and national levels, and for different periods of time, is related to psychological distress in different ways.

Although the obtained results seem promising, the current findings and methods have limitations. The main limitation of the research is its cross-sectional character. The obtained data only allowed fundamentally correlational analysis—the research should be followed up by studies using repeated measurements. In terms of COVID-19-related variables, we focused on the physical health variables, but it would also be worth controlling for variables that may influence psychological health, e.g., the extent of reductions in people’s social contact and their social isolation. Moreover, in terms of objective financial measures, we focused on income, savings, and financial liabilities, but it would also be worth tracing out the effects of wealth and control for respondents’ possessions. It should also be noted that the research sample was randomly selected and diversified in terms of gender, age, level of education, and place of residence; however, the sample was not fully representative of the Polish population. This should be taken into account when trying to generalize the results for the Polish population. For example, in our sample, 53.9% of respondents had higher education, while, in the Polish population, among people working professionally, this percentage is about 37.2% (data from 2020) [55]. Additionally, the percentage of women in our sample was significantly higher (64.5%) than the percentage of women in the Polish population of people working professionally (44.7%) [55].

Moreover, it should be considered that, across the world, there are huge cultural variations between Western; more individualistic nations (such as Poland); and other, more collectivistic cultures (e.g., Korea, Ghana, and Nigeria) [56,57], which may, for example, lead to various reactions to COVID-19-related changes in personal finances. In addition, Western countries differ significantly from non-Western nations on socioeconomic issues, such as income inequalities, social protection systems, and welfare benefits [58]. This means that our results may not be generalizable to other socioeconomic contexts, such as non-Western societies.

## 5. Conclusions

The present study adds to the current state of knowledge by showing that financial variables explain a significant portion of variance in PD over and above sociodemographic and COVID-19 health-related factors. The study confirmed the predictive role of three categories of financial factors: people’s objective financial situation, their subjective financial situation, and individual financial dispositions and showed the latter category to be the most important. Moreover, the study also identified individual financial variables which were capable of predicting people’s psychological distress during the pandemic, and showed the roles of financial variables to be similar in predicting the extent of people’s depression and anxiety symptoms. The current study found that depression and anxiety symptoms were higher among people with higher levels of materialism and a higher general propensity to take financial risks and short-term economic optimism, while psychological distress was lower among people with higher levels of financial security and long-term economic optimism. 

Our study provides valuable insights for individuals, therapists, and policy-makers by revealing the association between various financial factors (especially individual financial dispositions such as materialism, general propensity to take financial risks, and economic optimism) and psychological distress during the outbreak of the COVID-19 pandemic. For example, our results suggest that using various strategies to decrease materialistic values (such as encouraging people to focus more on intrinsic and self-transcendent values/goals [59]) may be helpful in reducing mental health problems during the COVID-19 pandemic.

## Figures and Tables

**Table 1:** Descriptive statistics for the socioeconomic variables.

Variable	Category	*n* (%)
Education	Primary education	79 (7.0)
	Secondary education	304 (26.8)
	Post-secondary	140 (12.3)
	Higher education—bachelor’s/engineer’s degree	149 (13.1)
	Higher education—master’s degree	463 (40.8)
Place of residence	Countryside	196 (17.3)
	Small town (<20,000 inhabitants)	130 (11.5)
	Medium town (20,000–99,000 inhabitants)	263 (23.2)
	Large city (100,000–500,000 inhabitants)	297 (26.2)
	Very large city (>500,000 inhabitants)	249 (21.9)
Household net income per capita	Less than PLN 1000	43 (3.8)
	PLN 1001–2000	174 (15.3)
	PLN 2001–3000	285 (25.1)
	PLN 3001–4000	197 (17.4)
	PLN 4001–5000	113 (10.0)
	More than PLN 5000	165 (14.5)
	Refused to answer *	158 (13.9)

* Taking into account the data on wages and inflation in the Polish population [46], it can be assumed that those who refused to answer the question about income were evenly distributed in each of the above groups.

**Table 2:** Results of two hierarchical regression analyses predicting psychological distress from categories of variables.

Dependent Variable	Independent Variable	*R*	*R* ^2^	Adjusted *R*^2^	*R*^2^ Change	*F* Change	*F* of the Model
Anxiety symptoms	Sociodemographic variables	0.223	0.050	0.046	0.050	12.658 ***	*F*(4, 972) = 12.658 ***
	COVID-19 health-related variables	0.254	0.065	0.059	0.015	7.851 ***	*F*(6, 970) = 11.175 ***
	Objective financial situation	0.303	0.092	0.082	0.027	5.820 ***	*F*(11, 965) = 8.892 ***
	Subjective financial situation	0.348	0.121	0.107	0.029	7.960 ***	*F*(15, 961) = 8.832 ***
	Individual financial dispositions	0.468	0.219	0.202	0.098	19.953 ***	*F*(21, 955) = 12.756 ***
Depression symptoms	Sociodemographic variables	0.228	0.052	0.048	0.052	13.361 ***	*F*(4, 972) = 13.361 ***
	COVID-19 health-related variables	0.256	0.065	0.060	0.013	6.848 ***	*F*(6, 970) = 11.297 ***
	Objective financial situation	0.308	0.095	0.084	0.029	6.262 ***	*F*(11, 965) = 9.176 ***
	Subjective financial situation	0.344	0.119	0.105	0.024	6.517 ***	*F*(15, 961) = 8.620 ***
	Individual financial dispositions	0.465	0.217	0.199	0.098	19.911 ***	*F*(21, 955) = 12.573 ***

*** *p* < 0.001.

**Table 3:** The results of two stepwise regression analyses predicting psychological distress from individual variables.

Dependent Variable	Statistically Significant Predictors	*Β*	*t*
Anxiety symptoms *F*(10, 966) = 25.878 *** R^2^ *=* 0.211, Adjusted *R^2^ =* 0.203	Materialism	0.233	7.751 ***
	Financial security	−0.134	−3.961 ***
	Gender ^a^	0.158	5.244 ***
	General propensity to take financial risks	0.124	3.885 ***
	Economic optimism (short-term)—national level	0.129	3.945 ***
	Perceived job security	−0.086	−2.756 **
	Risk grouping for severe COVID-19	0.092	3.162 **
	Age	−0.096	−3.102 **
	Economic optimism (long-term)—household level	−0.076	−2.289 *
	Amount of monthly commitments related to loans/credits	0.062	2.100 *
Depression symptoms *F*(8, 968) = 33.992 *** R^2^ *=* 0.204, Adjusted *R*^2^ *=* 0.198	Materialism	0.204	6.792 ***
	Financial security	−0.185	−5.874 ***
	Economic optimism (short-term)—national level	0.175	5.348 ***
	Gender ^a^	0.137	4.547 ***
	General propensity to take financial risks	0.137	4.301 ***
	Age	−0.131	−4.255 ***
	Risk grouping for severe COVID-19	0.094	3.211 ***
	Economic optimism (long-term)—household level	−0.103	−3.130 **

^a^ Coded: 1—female, 0—male; * *p* < 0.05; ** *p* < 0.01; *** *p* < 0.001.

## Data Availability

The complete data for all studies and the original materials used to conduct this research can be found on the Open Science Framework (OSF) website.

## Appendix A

**Table A1:** Descriptive statistics for the analyzed variables.

Variable	Percentage/Mean (*SD*) Total Sample *N* = 1135	Percentage/Mean (*SD*) Analyzed Sample *N* = 977
Gender (%)		
Male	35.5	36.2
Female	64.5	63.8
Age, mean (*SD*)	38.68 (11.59)	38.60 (11.64)
Place of residence (%)		
Countryside	17.3	16.8
Small town (<20,000 inhabitants)	11.5	12.3
Medium town (20,000–99,000 inhabitants)	23.2	23.2
Large city (100,000-500,000 inhabitants)	26.2	26.2
Very large city (>500,000 inhabitants)	21.9	21.5
Education (%)		
Primary education	7.0	7.4
Secondary education	26.8	27.1
Post—secondary	12.3	12.4
Higher education—bachelor’s/engineer’s degree	13.1	13.2
Higher education—master’s degree	40.8	39.9
COVID-19 experience (%)		
Yes	7.6	8.1
No	92.4	91.9
Risk group of severe COVID-19 (%)		
Yes	29.2	29.9
No	70.8	70.1
Monthly net income per capita (%)		
Less than PLN 1000	3.8	4.4
PLN 1001–2000	15.3	17.8
PLN 2001–3000	25.1	29.2
PLN 3001–4000	17.4	20.2
PLN 4001–5000	10.0	11.6
More than PLN 5000	14.5	16.9
Refuse to answer	13.9	-
Possession of savings		
Yes	74.8	72.6
No	25.2	27.4
Saving money before the COVID-19 pandemic		
Yes	73.2	71.8
No	26.8	28.2
Amount of monthly commitments related to loans/credits (%)		
No commitments	45.0	43.8
1–15% of household monthly income	14.0	14.5
16–30% of household monthly income	22.6	22.6
31–45% of household monthly income	9.2	9.1
46–50% of household monthly income	5.3	5.6
51–65% of household monthly income	1.4	1.6
66–80% of household monthly income	1.6	1.8
81–95% of household monthly income	0.4	0.3
More than 95% of household monthly income	0.5	0.5
Subjective assessment of current financial situation of household, mean (*SD*)	4.27 (1.31)	4.27 (1.32)
Subjective change of household financial situation, mean (*SD*)	3.41 (1.26)	3.41 (1.28)
Financial security, mean (*SD*)	3.53 (1.55)	3.54 (1.56)
Perceived job security (%)		
Yes	53.0	54.4
No	47.0	45.6
Materialism, mean (*SD*)	27.18 (6.40)	27.42 (6.36)
General propensity to take financial risks, mean (*SD*)	15.25 (8.47)	15.42 (8.58)
Economic optimism (short-term)—national level, mean (*SD*)	2.17 (1.47)	2.19 (1.49)
Economic optimism (long-term)—national level, mean (*SD*)	3.36 (1.67)	3.42 (1.69)
Economic optimism (short-term)—household level, mean (*SD*)	2.99 (1.38)	3.03 (1.40)
Economic optimism (long-term)—household level, mean (*SD*)	3.53 (1.42)	3.58 (1.46)
Anxiety symptoms, mean (*SD*)	14.63 (5.55)	14.71 (5.61)
Depression symptoms, mean (*SD*)	17.68 (6.45)	17.79 (6.51)

**Table A2:** Zero-order correlations for the analyzed variables.

	2	3	4	5	6	7	8	9	10	11	12	13	14	15	16	17	18	19	20	21	22
1	−0.26 ***	−0.02	0.10 **	0.01	−0.02	−0.09 **	−0.07 *	0.07 *	0.01	0.02	−0.07 *	−0.04	0.00	0.10 **	−0.10 **	−0.07 *	0.00	−0.06	−0.03	0.19 ***	0.17 ***
2		−0.07 *	0.02	−0.07 *	0.18 ***	0.07 *	0.00	−0.14 ***	0.08 *	−0.04	0.01	−0.03	−0.02	−0.16 ***	−0.13 ***	−0.03	−0.12 ***	−0.03	−0.13 ***	−0.16 ***	−0.19 ***
3			0.09 **	−0.02	−0.02	0.15 ***	0.00	0.01	−0.05	0.02	0.01	0.02	0.02	−0.04	−0.01	−0.04	−0.03	−0.02	0.01	−0.01	0.00
4				−0.06	0.03	0.16 ***	−0.01	0.15 ***	−0.02	0.06	0.02	0.00	0.03	−0.04	−0.11 **	−0.12 ***	−0.12 ***	−0.02	−0.11 **	−0.01	−0.02
5					0.07 *	0.06	−0.03	0.04	−0.01	0.06 *	0.05	0.04	−0.01	0.09 **	0.07 *	0.10 **	0.07 *	0.08 *	0.08 *	0.09 **	0.07 *
6						−0.01	−0.05	−0.01	0.03	−0.10 **	−0.10 **	−0.07 *	−0.05	−0.04	−0.02	−0.04	−0.04	−0.12 ***	−0.10 **	0.08 *	0.07 *
7							0.00	0.13 ***	0.01	0.34 ***	0.20 ***	0.22 ***	0.13 ***	0.00	0.00	−0.05	−0.02	0.11 **	0.07 *	−0.06	−0.08 *
8								0.10 **	−0.12 ***	0.10 **	0.10 **	0.13 ***	0.00	−0.10 **	−0.03	0.03	−0.03	0.06	0.01	−0.08 *	−0.07 *
9									−0.18 ***	0.27 ***	0.10 **	0.21 ***	0.08 *	−0.01	−0.06	−0.03	0.02	.09 **	0.07	−0.03	−0.05
10										−0.22 ***	−0.17 ***	−0.20 ***	−0.07 *	0.12 ***	0.04	−0.01	−0.01	−0.13 ***	−0.12 ***	0.130***	0.12 ***
11											0.60 ***	0.68 ***	0.28 ***	−0.03	0.06	0.21 ***	0.16 ***	0.42 ***	0.31 ***	−0.16 ***	−0.16***
12												0.66 ***	0.36 ***	−0.04	0.17 ***	0.34 ***	0.20 ***	0.60 ***	0.38 ***	−0.14 ***	−0.11 **
13													0.40 ***	−0.05	0.15 ***	0.26 ***	0.18 ***	0.53 ***	0.38 ***	−0.18 ***	−0.18 ***
14														0.00	0.02	0.07 *	0.10 **	0.35 ***	0.23 ***	−0.15 ***	−0.13 ***
15															0.22 ***	0.08 *	0.07 *	0.04	0.08 *	0.31 ***	0.28 ***
16																0.36 ***	0.21 ***	0.19 ***	0.20 ***	0.18 ***	0.20 ***
17																	0.51 ***	0.52 ***	0.37 ***	0.11 **	0.15 ***
18																		0.38 ***	0.62 ***	0.03	0.03
19																			0.63 ***	−0.06	−0.03
20																				−0.07 *	−0.06
21																					0.86 ***

* *p* < 0.05; ** *p* < 0.01; *** *p* < 0.001; 1—Gender (female (1) vs. male (0)); 2—Age; 3—Place of residence (city (1) vs. other (0)); 4—education (higher (1) vs other (0)); 5—COVID-19 experience; 6—Risk group of severe COVID-19; 7—Monthly net income; 8—Possession of savings; 9—Saving money before the COVID-19 pandemic; 10—Amount of monthly commitments related to loans/credits; 11—Subjective assessment of current financial situation of household; 12—Subjective change of household financial situation; 13—Financial security; 14—Perceived job security; 15—Materialism; 16—General propensity to take financial risks; 17—Economic optimism (short-term)—national level; 18—Economic optimism (long-term)—national level; 19—Economic optimism (short-term)—household level; 20—Economic optimism (long-term)—household level; 21—Anxiety symptoms; 22—Depression symptoms.

**Table A3:** Predictors of anxiety symptoms.

Predictors	*R*	*R* ^2^	Adjusted *R*^2^	Beta	*R*^2^ Change	*F* Change
Materialism	0.307	0.094	0.093	0.307 ***	0.094	101.495 ***
Materialism	0.348	0.121	0.120	0.299 ***	0.027	30.095 ***
Financial security				−0.165 ***		
Materialism	0.382	0.146	0.143	0.283 ***	0.024	27.585 ***
Financial security				−0.160 ***		
Gender				0.157 ***		
Materialism	0.416	0.173	0.170	0.240 ***	0.027	32.277 ***
Financial security				−0.187 ***		
Gender				0.177 ***		
General propensity to take financial risks				0.174 ***		
Materialism	0.427	0.183	0.179	0.238 ***	0.010	11.418 ***
Financial security				−0.209 ***		
Gender				0.181 ***		
General propensity to take financial risks				0.139 ***		
Economic optimism (short-term)—national level				0.108 ***		
Materialism	0.436	0.190	0.185	0.241 ***	0.008	9.037 **
Financial security				−0.171 ***		
Gender				0.181 ***		
General propensity to take financial risks				0.136 ***		
Economic optimism (short-term)—national level				0.106 ***		
Perceived job security				−0.095 **		
Materialism	0.444	0.197	0.191	0.244 ***	0.007	8.217 **
Financial security				−0.166 ***		
Gender				0.182 ***		
General propensity to take financial risks				0.136 ***		
Economic optimism (short-term)—national level				0.107 ***		
Perceived job security				−0.093 **		
Risk group of severe COVID-19				0.083 **		
Materialism	0.450	0.203	0.196	0.237 ***	0.006	7.049 **
Financial security				−0.167 ***		
Gender				0.162 ***		
General propensity to take financial risks				0.125 ***		
Economic optimism (short-term)—national level				0.109 ***		
Perceived job security				−0.093 **		
Risk group of severe COVID-19				0.096 ***		
Age				−0.081 **		
Materialism	0.456	0.208	0.200	0.240 ***	0.005	5.871 *
Financial security				−0.146 ***		
General propensity to take financial risks				0.159 ***		
Gender				0.128 ***		
Economic optimism (short-term)—national level				0.131 ***		
Perceived job security				−0.085 **		
Risk group of severe COVID-19				0.092 **		
Age				−0.090 **		
Economic optimism (long-term)—household level				−0.080 *		
Materialism	0.460	0.211	0.203	0.233 ***	0.004	4.410 *
Financial security				−0.134 ***		
Gender				0.158 ***		
General propensity to take financial risks				0.124 ***		
Economic optimism (short-term)—national level				0.129 ***		
Perceived job security				−0.086 **		
Risk group of severe COVID-19				0.092 **		
Age				−0.096 **		
Economic optimism (long-term)—household level				−0.076 *		
Amount of monthly commitments related to loans/credits				0.062 **		

* *p* < 0.05; ** *p* < 0.01; *** *p* < 0.001.

**Table A4:** Predictors of depression symptoms.

Predictors	*R*	*R* ^2^	Adjusted *R*^2^	Beta	*R*^2^ Change	*F* Change
Materialism	0.280	0.079	0.078	0.280 ***	0.079	83.187 ***
Materialism	0.325	0.105	0.104	0.272 ***	0.027	29.196 ***
Financial security				−0.164 ***		
Materialism	0.367	0.135	0.132	0.256 ***	0.030	33.221 ***
Financial security				−0.210 ***		
Economic optimism (short-term)—national level				−0.179 ***		
Materialism	0.398	0.159	0.155	0.239 ***	0.024	27.241 ***
Financial security				−0.208 ***		
Economic optimism (short-term)—national level				0.190 ***		
Gender				0.155 ***		
Materialism	0.421	0.177	0.173	0.207 ***	0.019	21.921 ***
Financial security				−0.219 ***		
Economic optimism (short-term)—national level				0.143 ***		
Gender				0.169 **		
General propensity to take financial risks				0.151 ***		
Materialism	0.432	0.187	0.182	0.196 ***	0.010	11.378 ***
Financial security				−0.222 ***		
Economic optimism (short-term)—national level				0.145 ***		
Gender				0.143 ***		
General propensity to take financial risks				0.137 ***		
Age				−0.103 ***		
Materialism	0.443	0.196	0.190	0.199 ***	0.010	11.490 ***
Financial security				−0.216 ***		
Economic optimism (short-term)—national level				0.147 ***		
Gender				0.140 ***		
General propensity to take financial risks				0.134 ***		
Age				−0.121 ***		
Risk group of severe COVID-19				0.099 ***		
Materialism	0.452	0.204	0.198	0.204 ***	0.008	9.800 **
Financial security				−0.185 ***		
Economic optimism (short-term)—national level				0.175 ***		
Gender				0.137 ***		
General propensity to take financial risks				0.137 ***		
Age				−0.131 ***		
Risk group of severe COVID-19				0.094 ***		
Economic optimism (long-term)—household level				−0.103 ***		

** *p* < 0.01; *** *p* < 0.001.

## References

[1] Cénat J.M., Blais-Rochette C., Kokou-Kpolou C.K., Noorishad P.G., Mukunzi J.N., McIntee S.E., Dalexis R.D., Goulet M.A., Labelle P.R. (2021). Prevalence of symptoms of depression, anxiety, insomnia, posttraumatic stress disorder, and psychological distress among populations affected by the COVID-19 pandemic: A systematic review and meta-analysis. Psychiatry Res..

[2] Lieberoth A., Lin S.Y., Stöckli S., Han H., Kowal M., Gelpi R., Chrona S., Tran T.P., Jeftić A., Rasmussen J. (2021). Stress and worry in the 2020 coronavirus pandemic: Relationships to trust and compliance with preventive measures across 48 countries in the COVIDiSTRESS global survey. R. Soc. Open Sci..

[3] Mirowsky J., Ross C.E. (2003). Social Causes of Psychological Distress.

[4] Horwitz A.V. (2007). Distinguishing distress from disorder as psychological outcomes of stressful social arrangements. Health London..

[5] Wang Y., Kala M.P., Jafar T.H. (2020). Factors associated with psychological distress during the coronavirus disease 2019 (COVID-19) pandemic on the predominantly general population: A systematic review and meta-analysis. PLoS ONE.

[6] Cai X., Hu X., Ekumi I.O., Wang J., An Y., Li Z., Yuan B. (2020). Psychological Distress and Its Correlates Among COVID-19 Survivors During Early Convalescence Across Age Groups. Am. J. Geriatr. Psychiatry.

[7] Ahmed M.Z., Ahmed O., Aibao Z., Hanbin S., Siyu L., Ahmad A. (2020). Epidemic of COVID-19 in China and associated Psychological Problems. Asian J. Psychiatry.

[8] Gambin M., Sękowski M., Woźniak-Prus M., Wnuk A., Oleksy T., Cudo A., Hansen K., Huflejt-Łukasik M., Kubicka K., Łyś A.E. (2021). Generalized anxiety and depressive symptoms in various age groups during the COVID-19 lockdown in Poland. Specific predictors and differences in symptoms severity. Compr. Psychiatry.

[9] Solomou I., Constantinidou F. (2020). Prevalence and Predictors of Anxiety and Depression Symptoms during the COVID-19 Pandemic and Compliance with Precautionary Measures: Age and Sex Matter. Int. J. Environ. Res. Public Health.

[10] Lei L., Huang X., Zhang S., Yang J., Yang L., Xu M. (2020). Comparison of Prevalence and Associated Factors of Anxiety and Depression Among People Affected by versus People Unaffected by Quarantine During the COVID-19 Epidemic in Southwestern China. Med. Sci. Monit..

[11] Olagoke A.A., Olagoke O.O., Hughes A.M. (2020). Exposure to coronavirus news on mainstream media: The role of risk perceptions and depression. Br. J. Health Psychol..

[12] Cao W., Fang Z., Hou G., Han M., Xu X., Dong J., Zheng J. (2020). The psychological impact of the COVID-19 epidemic on college students in China. Psychiatry Res..

[13] Wilson J.M., Lee J., Fitzgerald H.N., Oosterhoff B., Sevi B., Shook N.J. (2020). Job Insecurity and Financial Concern During the COVID-19 Pandemic Are Associated With Worse Mental Health. J. Occup. Environ. Med..

[14] Dijkstra-Kersten S.M., Biesheuvel-Leliefeld K.E., van der Wouden J.C., Penninx B.W., van Marwijk H.W. (2015). Associations of financial strain and income with depressive and anxiety disorders. J. Epidemiol. Community Health.

[15] Lorant V., Croux C., Weich S., Deliège D., Mackenbach J., Ansseau M. (2007). Depression and socio-economic risk factors: 7-year longitudinal population study. Br. J. Psychiatry.

[16] Orpana H.M., Lemyre L., Gravel R. (2009). Income and psychological distress: The role of the social environment. Health Rep..

[17] Bridges S., Disney R. (2010). Debt and depression. J. Health Econ..

[18] Fitch C., Hamilton S., Bassett P., Davey R. (2011). The Relationship between Personal Debt and Mental Health: A Systematic Review. Ment. Health Rev. Brighton.

[19] Wildman J. (2003). Income related inequalities in mental health in Great Britain: Analysing the causes of health inequality over time. J. Health Econ..

[20] Ferrie J.E., Shipley M.J., Stansfeld S.A., Smith G.D., Marmot M., Study W.I. (2003). Future uncertainty and socioeconomic inequalities in health: The Whitehall II study. Soc. Sci Med..

[21] Kahle L.R., Shoham A., Rose G., Smith M., Batra R. (2003). Economic versus Personal Future-Oriented Attitudes as Consumer Shopping Indicators. J. Euro Mark..

[22] Maner J.K., Schmidt N.B. (2006). The role of risk avoidance in anxiety. Behavioral.

[23] Muñiz-Velázquez J.A., Gomez-Baya D., Lopez-Casquete M. (2017). Implicit and Explicit Assessment of Materialism: Associations with Happiness and Depression. Pers. Individ. Dif..

[24] Kessler R.C. (1982). A disaggregation of the relationship between socioeconomic status and psychological distress. Am. Sociol. Rev..

[25] Link B.G., Lennon M.C., Dohrenwend B.P. (1993). Socioeconomic Status and Depression: The Role of Occupations Involving Direction, Control, and Planning. Am. J. Sociol..

[26] Kosidou K., Dalman C., Lundberg M., Hallqvist J., Isacsson G., Magnusson C. (2011). Socioeconomic status and risk of psychological distress and depression in the Stockholm Public Health Cohort: A population-based study. J. Affect. Disord..

[27] Brown S., Taylor K., Wheatley Price S. (2005). Debt and distress: Evaluating the psychological cost of credit. J. Econ. Psychol..

[28] Skapinakis P., Weich S., Lewis G., Singleton N., Araya R. (2006). Socio-economic position and common mental disorders. Longitudinal study in the general population in the UK. Br. J. Psychiatry.

[29] Jenkins R., Bhugra D., Bebbington P., Brugha T., Farrell M., Coid J., Fryers T., Weich S., Singleton N., Meltzer H. (2008). Debt, income and mental disorder in the general population. Psychol. Med..

[30] Drentea P. (2000). Age, debt and anxiety. J. Health Soc. Behav..

[31] Gardner J., Oswald A.J. (2007). Money and mental wellbeing: A longitudinal study of medium-sized lottery wins. J. Health Econ..

[32] Pearlin L.I., Johnson J.S. (1977). Marital status, life-strains and depression. Am. Sociol. Rev..

[33] O’Neill B., Sorhaindo B., Xiao J.J., Garman E.T. (2005). Financially Distressed Consumers: Their Financial Practices, Financial Well-being, and Health. J. Fin. Couns. Plan..

[34] Gutter M., Copur Z. (2011). Financial Behaviors and Financial Well-Being of College Students: Evidence from a National Survey. J. Fam. Econ. Issues.

[35] Richins M.L., Dawson S. (1992). A Consumer Values Orientation for Materialism and Its Measurement: Scale Development and Validation. J. Consum. Res..

[36] Kasser T., Ryan R.M. (1993). A dark side of the American dream: Correlates of financial success as a central life aspiration. J. Pers. Soc. Psychol..

[37] Ryan R.M., Chirkov V.I., Little T.D., Sheldon K.M., Timoshina E., Deci E.L. (1999). The American Dream in Russia: Extrinsic Aspirations and Well-Being in Two Cultures. Pers. Soc. Psychol. Bull..

[38] Wang R., Liu H., Jiang J., Song Y. (2017). Will Materialism Lead to Happiness? A Longitudinal Analysis of the Mediating Role of Psychological Needs Satisfaction. Pers. Individ. Dif..

[39] Dittmar H., Bond R., Hurst M., Kasser T. (2014). The relationship between materialism and personal well-being: A meta-analysis. J. Pers. Soc. Psychol..

[40] van Giesen R.I., Pieters R. (2019). Climbing out of an Economic Crisis: A Cycle of Consumer Sentiment and Personal Stress. J. Econ. Psychol..

[41] Eisenberg A., Baron J., Seligman M.E.P. Individual Differences in Risk Aversion and Anxiety. https://www.sas.upenn.edu/~baron/papers.htm/amyold.html.

[42] Kuhnen C.M., Knutson B. (2011). The Influence of Affect on Beliefs, Preferences, and Financial Decisions. J. Fin. Quant. Anal..

[43] Raghunathan R., Pham M.T. (1999). All Negative Moods Are Not Equal: Motivational Influences of Anxiety and Sadness on Decision Making. Organ. Behav. Hum. Decis. Process..

[44] Nicholson N., Soane E., Fenton-O’Creevy M., Willman P. (2005). Personality and Domain-specific Risk Taking. J. Risk Res..

[45] Faul F., Erdfelder E., Lang A.G., Buchner A. (2007). G*Power 3: A flexible statistical power analysis program for the social, behavioral, and biomedical sciences. Behav. Res. Methods.

[46] Statystyczny G.U. (2019). Dochody i Warunki Życia Ludności Polski–Raport z Badania EU-SILC 2018.

[47] Spitzer R.L., Kroenke K., Williams J.B., Löwe B. (2006). A brief measure for assessing generalized anxiety disorder: The GAD-7. Arch. Intern. Med..

[48] Kroenke K., Spitzer R.L., Williams J.B. (2001). The PHQ-9: Validity of a brief depression severity measure. J. Gen. Intern. Med..

[49] Richins M.L. (2004). The Material Values Scale: Measurement Properties and Development of a Short Form. J. Consum. Res..

[50] Blais A.-R., Weber E.U. (2006). A Domain-Specific Risk-Taking (DOSPERT) scale for adult populations. Judgm. Decis. Mak..

[51] Bendau A., Plag J., Kunas S., Wyka S., Ströhle A., Petzold M.B. (2021). Longitudinal changes in anxiety and psychological distress, and associated risk and protective factors during the first three months of the COVID-19 pandemic in Germany. Brain Behav..

[52] Bell V., Wade D. (2021). Mental health of clinical staff working in high-risk epidemic and pandemic health emergencies a rapid review of the evidence and living meta-analysis. Soc. Psychiatry Psychiatr. Epidemiol..

[53] Ritchie H., Ortiz-Ospina E., Beltekian D., Mathieu E., Hasell J., Macdonald B., Giattino C., Appel C., Rodés-Guirao L., Roser M. Coronavirus Pandemic (COVID-19). https://ourworldindata.org/coronavirus.

[54] Xie X.-F., Stone E., Zheng R., Zhang R.-G. (2011). The ‘Typhoon Eye Effect’: Determinants of Distress during the SARS Epidemic. J. Risk Res..

[55] Statystyczny G.U. (2021). Rocznik Statystyczny Rzeczypospolitej Polskiej.

[56] Hofstede G. (1980). Culture’s Consequences: International Differences in Work-Related Values.

[57] Ibrahim S., Maxwell S.R., Blair S.L. (2015). A binary model of broken home: Parental death-divorce hypothesis of male juvenile delinquency in nigeria and ghana. Contemporary Perspectives in Family Research.

[58] International Labour Office (2021). World Social Protection Report 2020–22: Social Protection at the Crossroads-in Pursuit of a Better Future.

[59] Kasser T. (2016). Materialistic values and goals. Annu. Rev. Psychol..

